# Supplementary figures and images for: Transcriptomic profiling of nematode parasites surviving vaccine exposure
Source: Int J Parasitol. 2018 Apr;48(5):395–402. doi: 10.1016/j.ijpara.2018.01.004 (PMC5909036; doi:10.1016/j.ijpara.2018.01.004)

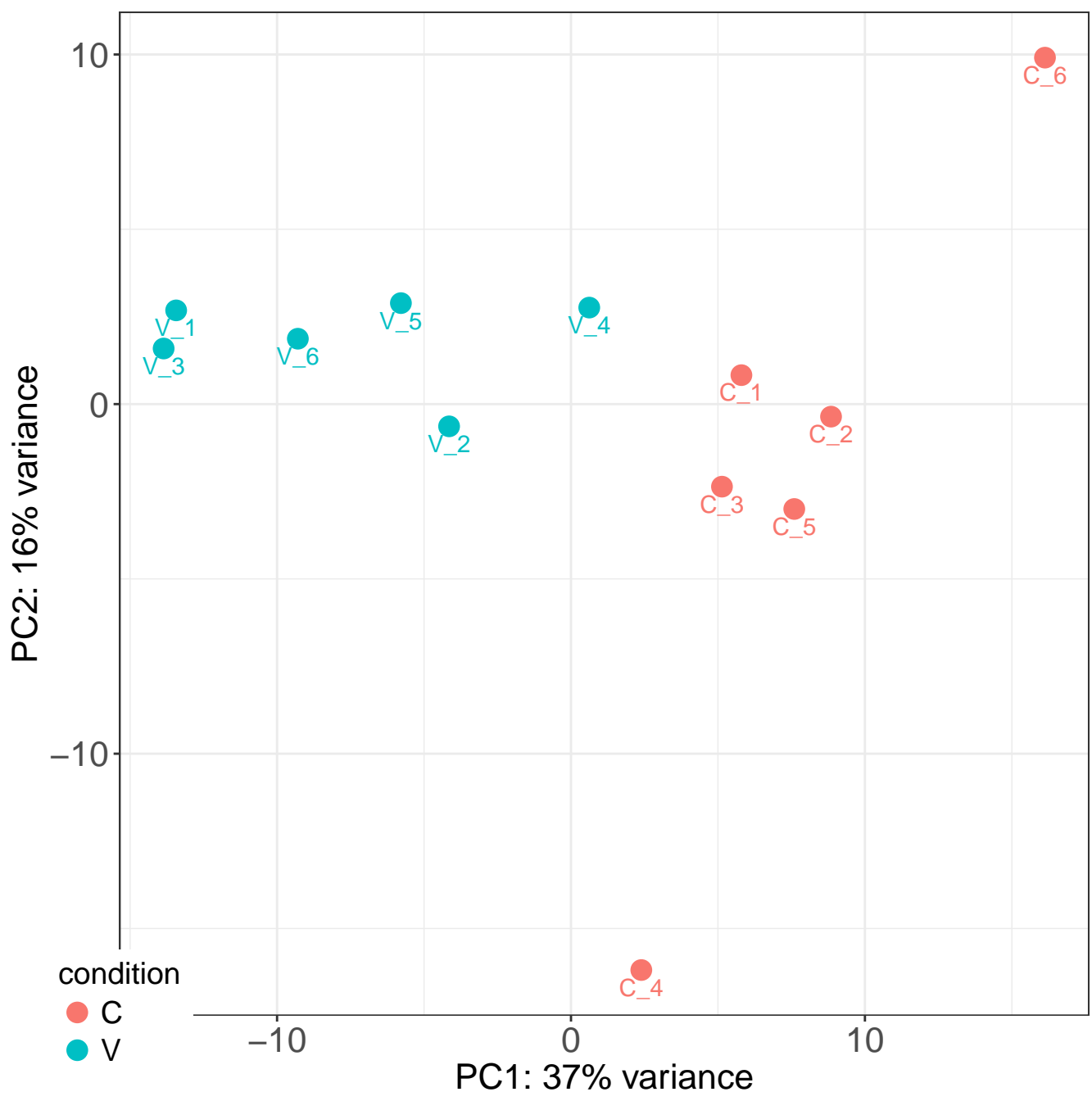

Supplement: Supplementary Fig. S1 — Principal component analysis (PCA) of transcript counts measured in worms collected from vaccinated or control sheep. PCA is a dimensionality reduction method that makes use of transcript counts to define a new set of unrelated components. Coordinates of every pool of worms considered for analysis are plotted against the first two components and correlate with similarities between pools. The first PCA axis explains 36% of total variance and relates to differences between the two considered experimental groups, i.e. worms exposed to the vaccine response (V) or the control group (C). [file mmc1.pdf]

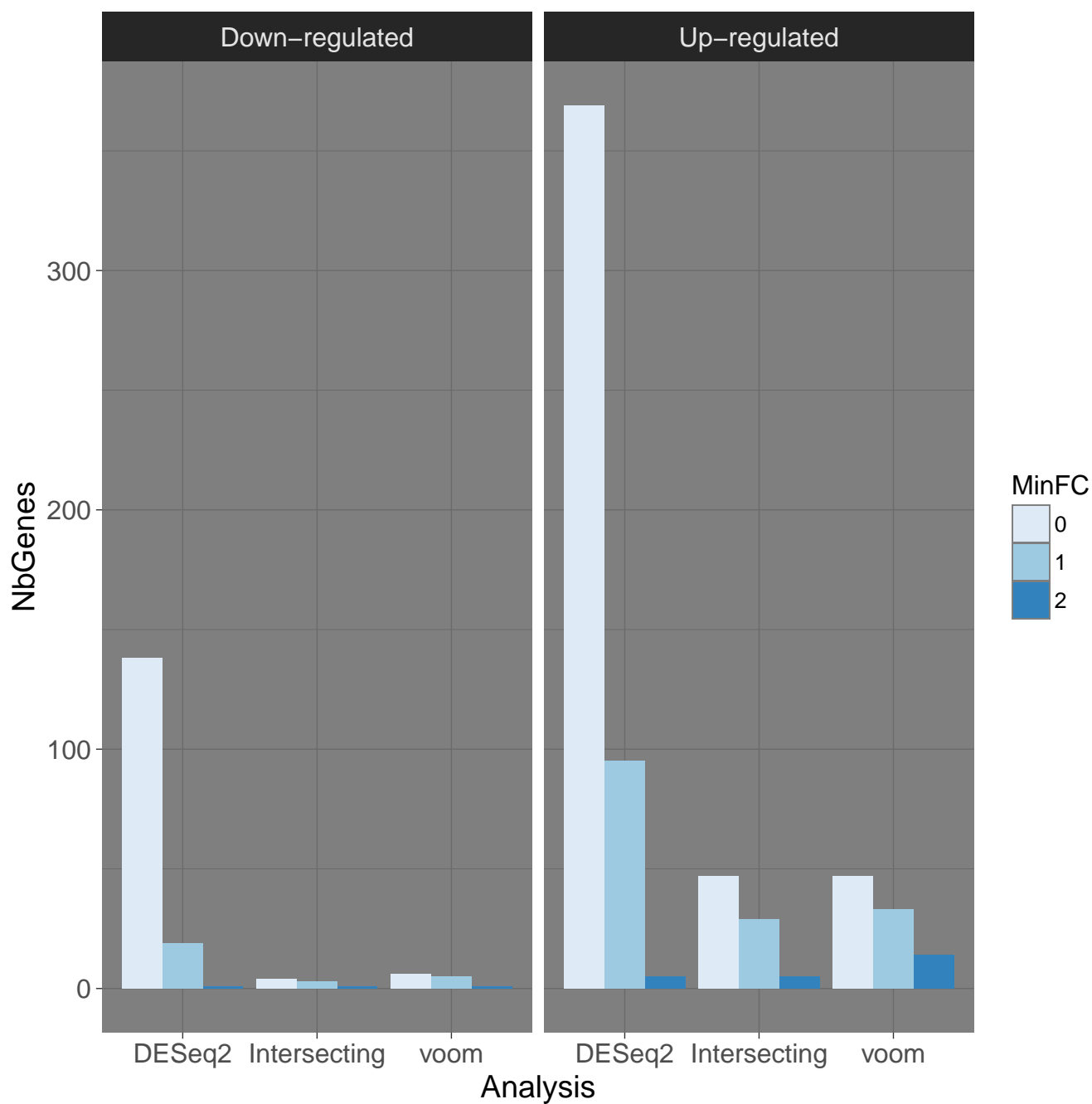

Supplement: Supplementary Fig. S2 — Number of differentially expressed (DE) genes found by each of the two implemented methods (DESeq2, voom). Total number of significantly DE genes found by at least one of the two methods or both (intersecting) are plotted according to their regulation pattern, i.e up- or down-regulated in the vaccine survivors, to their estimated fold change (FC), i.e. log2FC > 2, 1 or 0. [file mmc2.pdf]
